# Designing and Implementing TeleBehavioral Health Training to Support Rapid and Enduring Transition to Virtual Care in the COVID Era

**DOI:** 10.1007/s41347-022-00286-y

**Published:** 2022-12-14

**Authors:** Bradford L. Felker, Cara B. Towle, Ingrid K. Wick, Melody McKee

**Affiliations:** 1 Department of Veterans Affairs, Puget Sound Healthcare System, Seattle, WA USA; 2 Department of Psychiatry and Behavioral Sciences, University of Washington, Seattle, WA USA; 3 Behavioral Health Institute Harborview, University of Washington, Seattle, WA USA; 4 Workforce and Policy Innovation Center, Behavioral Health Training, University of Washington/UW Medicine, Seattle, WA USA; 5 Harborview Medical Center, Behavioral Health Institute, University of Washington/UW Medicine, Seattle, WA USA; 6 Telepsychiatry, Psychiatry and Behavioral Sciences, University of Washington/UW Medicine, Seattle, WA USA

**Keywords:** TeleBehavioral Health, Training, Implementation science

## Abstract

As telebehavioral health continues to advance and become part of routine care, there is a need to develop effective training methods. While a consensus on how to best train telebehavioral health has not yet been achieved, this commentary will describe how evidence-based implementation strategies were used to develop a framework to create and implement a telebehavioral health training program that is relevant and enduring for a given audience. Evidence-based implementation strategies included the PARiHS criteria which were used to organize the project. Re-AIM criteria was used to organize chosen outcome measures. Important partnerships were formed to help support infrastructure as well as regional and national reach. A series of Plan-Do-Study-Act loops were used to inform progressive training series. Since April 2020, the Behavioral Health Institute has developed and offered 6 unique telebehavioral health training series, employing both webinar and online formats, and addressing core components as well as more advanced concepts. These series have provided over 19,100 accredited continuing education hours of training through June 2022, to almost 3000 unique learners via webinar and nearly 6800 unique online learners, across 45 states. Evaluations rated these trainings as high quality, relevant, and that material would likely be implemented. Feedback from attendees was considered vital in series planning. This commentary discusses how evidence-based implementation strategies can be used to create a framework upon which to base a training program for health care providers. An example is given on how this framework was used to create successful, relevant, and enduring telebehavioral health training.

## Objectives

Telebehavioral health, primarily the use of synchronous video teleconference technology (exclusive of audio-only telephone care) to deliver behavioral health care services, has been shown to be effective and its use was steadily increasing prior to the COVID-19 public health emergency (Hilty et al., 2020; Chakrabarti, 2015; Barnett et al., 2018; Patel et al., 2020). The COVID-19 pandemic caused a dramatic shift in the delivery of care as providers adopted the use of telebehavioral health modalities and began to form opinions on how to best use these modalities (Rosen et al., 2021; Connolly et al., 2021; Lindsay et al., 2020)

There has been steady growth in the way telebehavioral health is taught (Rousmaniere et al., 2014; Hilty et al., 2020; Crowfoot & Prasad, 2017; Caver et al., 2020; Hilty et al., 2020; Jordan & Shearer, 2019; Traube et al., 2021; Felker et al., 2021) and a variety of organizations offer resources, training, and conferences focused on telehealth and telemental health. However, to date, a consensus on how telehealth should be taught has not emerged (Hilty et al., 2015a, b; Edirippulige & Armfeld, 2017; Chike-Harris et al., 2021; Hilty 2020). Creating relevant curriculum has been difficult given the rapid expansion and changes related to telebehavioral health technologies, changing policies and regulations, new telebehavioral health modalities (e.g., asynchronous services, platforms powered by artificial intelligence), the different skill sets needed by providers and administrators, and challenges related to the “digital divide” (Mitchell et al., 2019; Vassilakopoulou & Hustard, 2021; National Digital Inclusion Alliance, 2020).

At the onset of the COVID-19 pandemic in early April 2020, the Washington State Health Care Authority (HCA) approached the Behavioral Health Institute (BHI) at the University of Washington (UW)/Harborview Medical Center to urgently create and implement a telebehavioral health training curriculum to support behavioral health care providers across Washington state, as they quickly pivoted to providing telebehavioral health care. The BHI is a newly established innovation center that, in partnership with the UW Department of Psychiatry and Behavioral Sciences, had established priority programs to include improving care for youth and young adults with early psychosis, an Urgent Care Walk-in clinic, Behavioral Health Training, and expanded Digital and Telebehavioral Health services. In response to the HCA request, the BHI has since created 6 unique telebehavioral health training series, using webinar and online formats.

The objective of this commentary is to describe how evidence-based implementation strategies were used to create, implement, evaluate, and update an easily accessed practical clinical video telebehavioral health curriculum designed for the busy frontline mental health provider.

## Methods

The COVID-19 pandemic had essentially forced providers and their clinics to rapidly transition to virtual care, incorporating unfamiliar technology and modalities, as well as fast-moving regulatory changes, while responding to an expanding need for behavioral health services. The HCA asked the BHI to provide training in how to deliver professional telebehavioral health services, while abiding by a rapidly evolving regulatory landscape. To accomplish this task, the BHI assembled a project team with digital and telebehavioral health expertise. Given the complexity of this task, the project team envisioned a quality improvement project and realized that using a series of implementation strategies and methodologies would be needed to help guide proper implementation and evaluation of this training initiative.

Although there were many different educational topics and delivery models for implementing a telebehavioral health curriculum, the BHI team ultimately decided to use a traditional didactic lecture format delivered over Zoom in response to this imperative from the HCA for rapid training. The didactic lecture series content would focus on what the BHI team agreed were foundational practical information and core elements for completing a professional video encounter and would target frontline clinicians and administrators across the state. The project team also agreed that an evaluation system was needed to understand the impact and to plan strategically for future training series.

To provide a relevant framework and guide implementation, the team organized the telebehavioral health training series using criteria set forth in Promoting Action on Research Implementation in Health Services (PARiHS) (Kitson et al., 2008; Stetler et al., 2011). PARiHS criteria can be useful in further organizing a project in terms of context, evidence, and facilitation. This framework also facilitates communication about the project with leadership and other stakeholders.

### PARiHS Context

The COVID-19 pandemic compelled a rapid shift to providing telebehavioral health care, with an accompanying need to provide foundational training to help frontline providers complete a professional virtual clinical encounter. The target audience for this training program was community mental/behavioral health clinics, providers, and administrators who had limited telebehavioral health knowledge, experience, or skills. There was a need to create a broad-based curriculum that was practical, provided foundational telebehavioral health clinical skills training as well as applicable regulatory information, and could easily be disseminated and attended.

### PARiHS Evidence

The literature is clear that video telemental health is effective (Hubley et al., 2016). However, for it to be effective, providers need to understand how to properly implement telebehavioral health in a clinical setting (Edirippulige & Armfield, 2017; Meyer et al., 2020). To stay focused on developing a practical training series for the frontline provider, invited instructors were asked to develop lectures that were tailored to the beginner and practical for a busy provider or administrator, and information needed to be grounded in evidence-based literature with references provided.

### PARiHS Facilitation

To best *facilitate* the implementation of these training series, the project team partnered with organizations, as described below, to help establish infrastructure to support administrative tasks such as the creation, implementation, and evaluation of participant surveys, and advertising the series through extensive membership networks. In addition, to promote attendance for busy frontline providers, both a webinar series and online training series that can be accessed anytime were created.

In addition, the project team realized that ongoing *evidence* would be needed to better understand the value, feasibility, and sustainability of ongoing telebehavioral health trainings. To better understand and structure outcomes, the project team organized outcomes using the RE-AIM Framework which uses the following domains: reach, effectiveness, adoption, implementation, and maintenance (Gaglio et al., 2013; Glasgow et al., 2001).

To evaluate reach, effectiveness, and adoption, the project team designed a registration form and a post-lecture evaluation survey for each session. For reach, data regarding unique attendees, total participations, and geographic impact was tracked. For effectiveness, participant surveys were added to address quality of the presentation, whether objectives were met, and usefulness to their practice. For adoption, participants were surveyed as to the trainings value, relevance, utility, and impact. For implementation, the project team did not use a specific measure. Instead, it monitored attendance and feedback from the measures listed above to determine the need and subject matter for potential subsequent training. For maintenance, the project team used a series of Plan-Do-Study cycles to guide the development of subsequent training series (Crowfoot & Prasad, 2017, https://www.ahrq.gov/health-literacy/improve/precautions/tool2b.html). Prior to developing each subsequent training series, the team evaluated feedback from participants to identify emerging themes and design a subsequent series to best meet expressed needs. In so doing, the team sought to maintain relevant training series to best meet the needs of its participants.

The project team partnered with various organizations to *facilitate* implementation of the training series. The initial webinar series, the Telehealth Provider Forum (Forum), was offered April–July 2020. The Northwest Mental Health Technology Transfer Center (NWMHTTC) (mhttcnetwork.org), a SAMHSA-funded network for technology transfer and dissemination of evidence-based mental health care, provided a Zoom webinar platform and support for registration and evaluation for the initial eight forum sessions. Thereafter, the BHI project team sought participant feedback using SurveyMonkey, and engaged the UW Telehealth Services for Zoom webinar, registration, and technical support. For the subsequent TeleBehavioral Health (TeleBH) 101, 201, and 301 series, the BHI project team has utilized REDCap (project-redcap.org) to manage evaluation, to track attendance, and to distribute and evaluate participant surveys described below.

In 2020, Washington state passed new legislation effective January 1, 2021, mandating that all providers offering telehealth care, except MDs and DOs, complete a certificated Telehealth Training (SSB 6061). TeleBH 101 session #1 was designed to meet that mandate and was offered as a webinar in early January 2021. In an effort to make that session, and all the TeleBH training sessions, more broadly available, the BHI team created an online version of the training series. To support and promote the online TeleBH training series, the project team partnered with the Northwest Regional Telehealth Resource Center (nrtrc.org). As one of 14 HRSA-funded telehealth resource centers, the NRTRC serves a seven-state region (AK, WA, OR, MT, ID, WY, UT) to advance the development, implementation, and integration of telehealth. The NRTRC hosts the online TeleBH training series on their Canvas Learning Management System (https://nrtrc.catalog.instructure.com). TeleBH 101 session #1 was launched in December 2020 to meet the newly mandated telehealth training requirement, with sessions #2–6 launched in March 2021. With the NRTRC’s support, Canvas has also been used to collect, aggregate, analyze, and report registration and evaluation data from the online training participants. Information about these training series was subsequently shared with the National Consortium of Telehealth Resource Centers to further promote this series nationally.

To further encourage attendance, the project team obtained accreditation for category 1 continuing medical education (CME) and the National Association of Social Workers (NASW) credit for attending each TeleBH training webinar or online session. In addition, a personalized certificate of completion is available at no cost via REDCap for webinar attendees and Canvas for online learners. Several provider types, such as psychologists, can use this certificate of completion to claim continuing education credit to meet licensure requirements.

As the TeleBehavioral Health training series has grown, BHI staff creates and manages all outreach and communications through its own growing listserv and collaborates with these partner organizations to share the TeleBH training series via listservs, newsletters, and websites. The BHI website has become an essential component of the TeleBH training series, both for outreach and for access to information, registration, past presentations, and continuing education and accreditation information (https://bhinstitute.uw.edu/training-workforce-policy/training/)).

## Results

Since April 2020, the BHI has developed and delivered 6 unique telebehavioral health training series, employing both real-time interactive webinar and online self-paced formats, and addressing core components as well as more advanced concepts:

- Telehealth Provider Forum webinar series, varied cadence, April–July 2020
- TeleBehavioral Health 101 webinar series, weekly, January–February 2021
- TeleBehavioral Health 201 webinar series, monthly, October 2020–September 2021
- TeleBehavioral Health 301 webinar series, monthly, January–December 2022
- TeleBehavioral Health 101 online series, December 2020 and ongoing
- TeleBehavioral Health 201 online series, June 2022 and ongoing

**Fig. 1:**
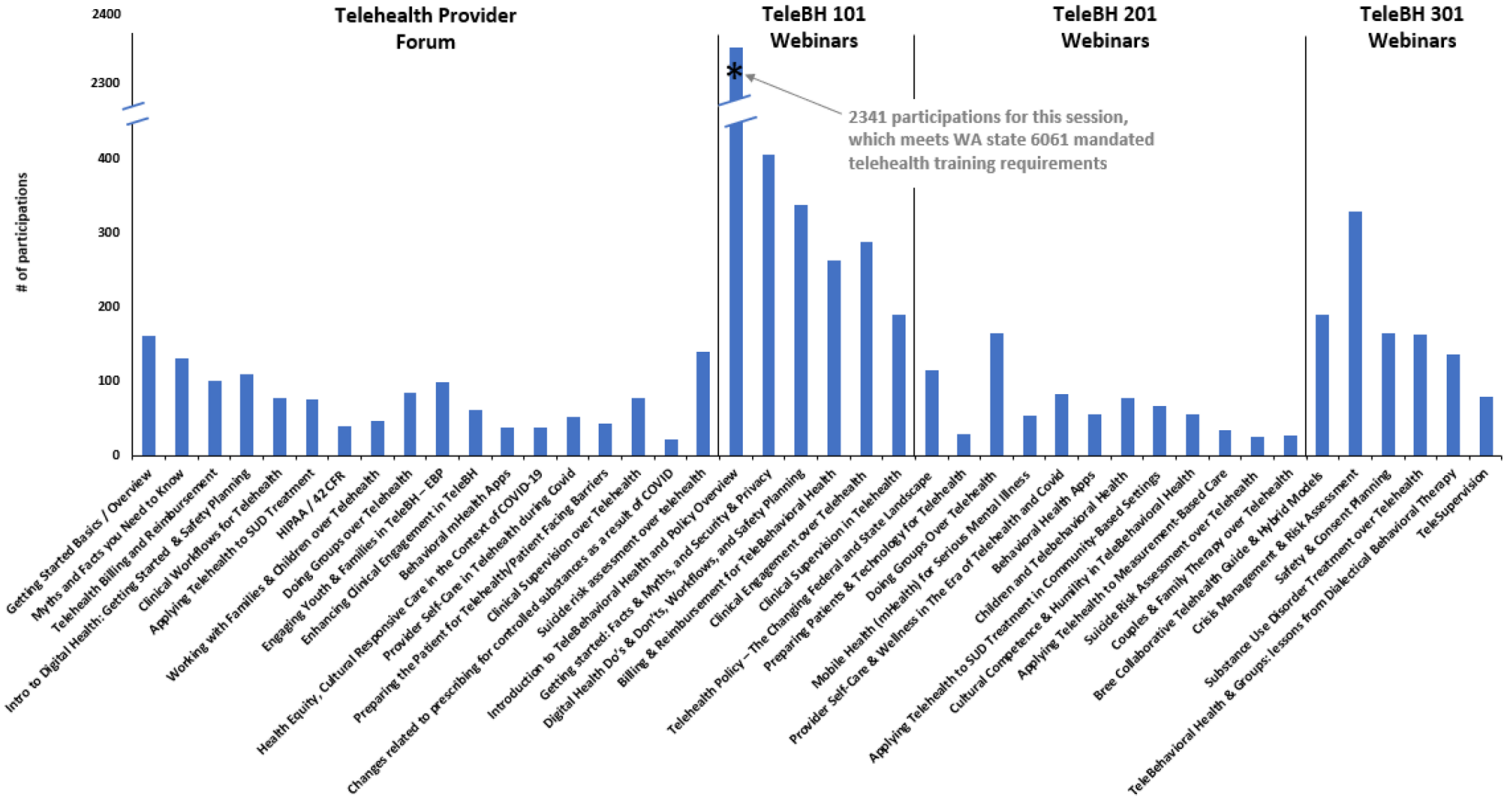
Telebehavioral health training webinar participations

**Fig. 2:**
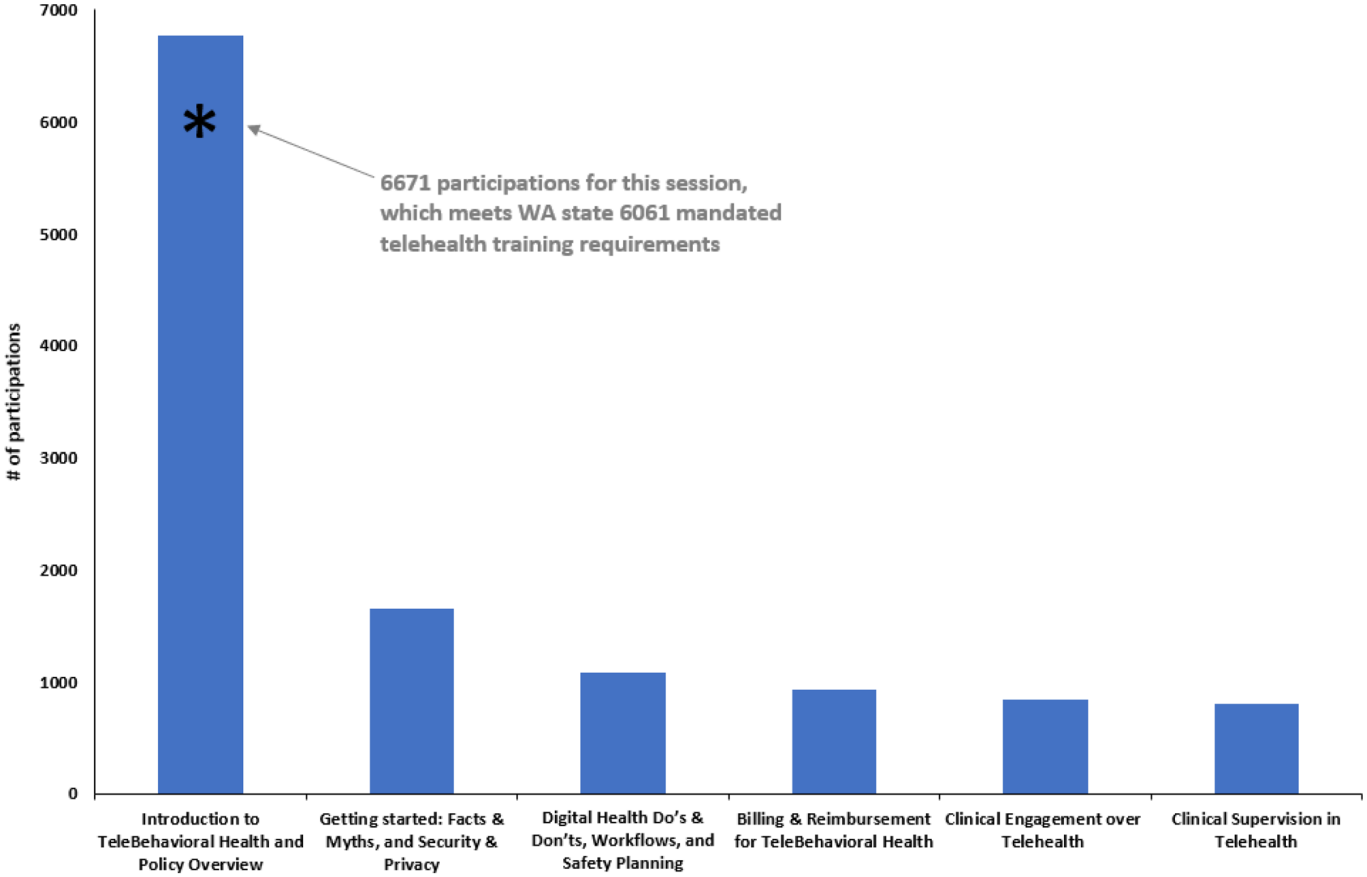
Telebehavioral health 101 online series participations

Please refer to Figs. 1 and 2 to see all components of the trainings.

Each subsequent training series was developed using the PDSA cycle approach and this ongoing evaluation data was used to inform priorities and structure for each subsequent series. For the Telehealth Provider Forum, evaluation only assessed “most useful information,” and suggestions for future topics and for support/training/materials, all with open text fields. While developing the TeleBH set of series, the project team developed a more extensive set of evaluation questions to better assess quality, impact, and ongoing needs, using drop-down menus when possible and as appropriate to more effectively aggregate data. Evaluation data collected through June 2022, as presented below, includes the Telehealth Provider Forum, the TeleBH 101 and 201 webinar series, and the TeleBH 101 online series. Evaluation data is still being collected on the 301 webinar series and the just-launched 201 online series.

As noted, outcomes were organized using the RE-AIM Framework domains: reach, effectiveness, adoption, implementation, and maintenance.

For reach, data regarding the number of providers was tracked across all six of the training series. The unique number of trainees has been tracked, as well as the total number of participations. For example, if one trainee attended six sessions, they would be counted as one unique trainee, but as six participations.

For reach, the total number of participations through June 2022, for all webinar and online training series, was 19,141, with at least 2655 unique learners attending webinars and nearly 6800 unique learners taking online courses. Table 1 provides participation details for each of these series; Fig. 1 provides participation details specific to each session of the telebehavioral health training webinars series, and Fig. 2 provides participation details specific to each session of the telebehavioral health training online series. As can be seen, greatest attendance was for the TeleBH 101 session #1 “Introduction to TeleBehavioral Health,” which was specifically designed to meet criteria for the state-mandated telehealth training described above. The number of unique learners across all series was not determined due to varied attendance tracking platforms. It is assumed that TeleBH 101 webinar attendees have taken subsequent webinar sessions, though overlap in participation between TeleBH 101 webinar attendees and TeleBH 101 online series is less likely given the identical curriculum and content.

The types of providers who participated included a broad variety of degrees, such as physicians, nurse practitioners and nurses, psychologists, physician assistants, mental health counselors, therapists, social workers, substance use disorder professionals, peer counselors, pharmacists, and billing specialists, with titles ranging from student to executive director. Participants were employed in a range of settings, including academic, non-academic, and critical access hospitals; multispecialty clinics; community clinics; Federally Qualified Health Centers; and free-standing/private clinics. Unfortunately, software and design limitations did not allow us to quantify and aggregate data related to credentials, professional titles or roles of trainees, or their professional settings. This issue will be addressed for future training series.

In total, 3832 evaluations were completed for participations in TeleBH 101 and 201 webinars. Among them, 26% noted providing services to American Indian and/or Alaska Natives, 22% noted providing services to Veterans, and 33% noted providing services to other specials populations, described as including other ethnic or linguistic groups, immigrants, refugees, homeless, low income, LGBTQ, physically impaired, developmentally disabled, incarcerated, pediatric/youth, and geriatric populations. Also, 22% noted providing services in rural settings, 28% in suburban settings, and 50% in urban settings.

**Fig. 3:**
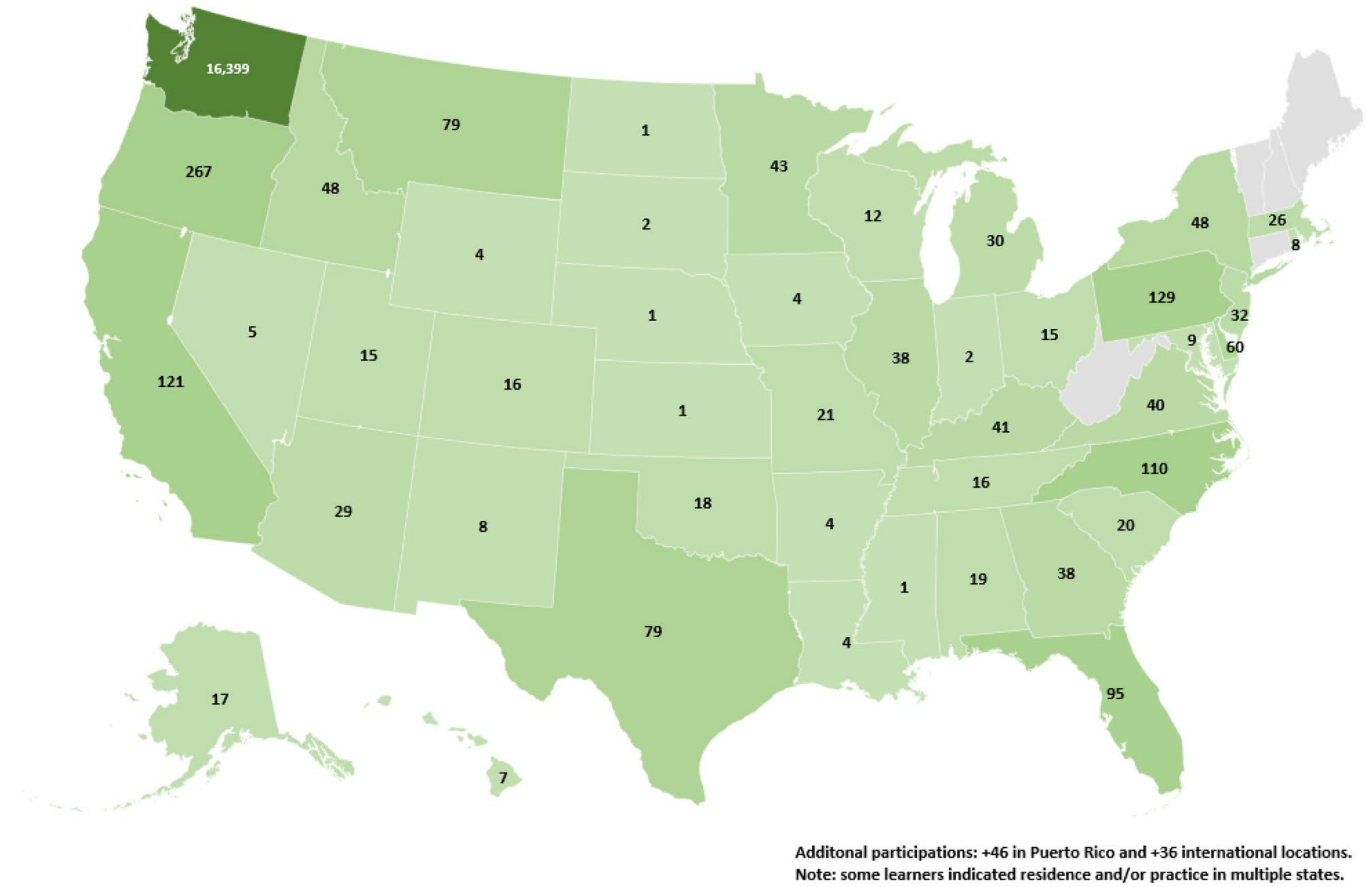
Geographic distribution of participations: inclusive of forums, webinars, and online sessions

By establishing partnerships with the supporting organizations listed above, these training series achieved national reach. Figure 3 illustrates impact in each state and beyond, through June 2022, for the Forum and the TeleBH 101, 201, and 301 webinars, and the TeleBH 101 online series.

For effectiveness, the initial Telehealth Providers Forum obtained limited feedback, mainly through open text field responses that focused on suggestions for future topics and training materials. The subsequent training series used a more detailed evaluation system, placing a greater emphasis on the quality of the presentation, if objectives were met, and usefulness to the trainees’ practice. Through June 2022, a total 12,908 evaluations were submitted for TeleBH 101 and 201 webinar series and TeleBH 101 online series.

Effectiveness data for these series has shown high ratings in terms of quality of presentation. TeleBH 101 and 201 webinars were rated 60.2% as excellent and 38.8% as satisfactory; TeleBH 101 online courses were rated 32.9% as excellent, 30% as very good, 27.6% as good, and 8.5% as satisfactory. Across all three series, 81.9% felt that activity objectives had been met.

For adoption, participants were surveyed as to training value, relevance, utility, and impact, and whether they would recommend to colleagues. When asked if the session was relevant to their work, 66.2% of participants in TeleBH 101 and 201 webinars rated the trainings as “very relevant,” and 32.4% rated the trainings as “somewhat relevant.” Of TeleBH 101 online participants, 43.3% rated the trainings as “very relevant,” with 38.7% rating it as “relevant,” and 32.6% rating it as “somewhat relevant.” The webinars and online courses were evaluated in a uniform manner for the remainder of the questions. Across both TeleBH 101 and 201 webinars and TeleBH 101 online series, when asked whether the information presented enhanced current knowledge, 69.2% answered “yes” and 25.8% answered “somewhat.” When asked whether the presentations provided new ideas or information they expect to use, 62.2% answered “yes” and 27% answered “somewhat.” When asked if the information presented addressed competencies in their specialty, 65.4% answered “yes” and 27% answered “somewhat.” When asked whether they intended to make changes or apply learning to their practice as a result of attending this activity, 54.4% answered “yes” and 25.8% answered “somewhat.” Finally, when asked if they would recommend the session to colleagues, 73.2% answered “yes,” and 19.5% answered “somewhat.”

As noted, the project team did not use a specific measure for implementation. Instead, it monitored attendance and feedback from the measures listed above to determine the need and subject matter for potential subsequent training series.

In terms of maintenance, the project used a series of PDSA cycles to design subsequent training series to best fit the needs of the participants. During the 18-session Telehealth Provider Forum webinar series, audience feedback data was analyzed immediately after each session to inform ongoing planning and content needs, which formed the initial PDSA cycle. The initial goal of rapidly providing foundational skills to complete a virtual encounter was achieved, but feedback indicated that there was ongoing need to provide basic skills and introduction of more advanced skills. In addition, to inform future series, the BHI team distributed two BHI surveys across the state of Washington—a telehealth provider survey and a telehealth patient survey.

These results informed the second PDSA cycle and led to what would become the TeleBehavioral Health 101 (TeleBH 101) series. This six-part series covered foundational information for understanding important policies and techniques for professional telebehavioral health encounters, and session #1 of TeleBH 101 intentionally met the state-mandated SSB 6061 telehealth training requirement. Also, based on feedback learned from the first PDSA cycle, access to the trainings was increased by converting the TeleBH 101 webinars to an online self-paced course on an LMS platform that could be accessed anytime.

The third PDSA cycle was informed by previous evaluation data and led to the content development for the TeleBH 201 series, which included more detailed presentations on policy, on providing telebehavioral health care to specific populations, and on using various digital tools in the clinical encounter. In a continued effort to increase access to these trainings, the BHI team allowed learners to complete these trainings in any order and without any pre-requisite training requirements. In other words, though recommended to complete the series in order, participants can pick and choose sessions in a way that is most valuable for them.

Ongoing evaluation data, and a new survey among trainees assessing future topics and preferences related to cadence, schedule, training platform, and modality, informed the fourth PDSA, leading to TeleBH 301, currently being offered as a webinar series, and the launch in late June 2022 of the TeleBH 201 online series.

Further information about these series is provided below.

## Discussion

Using a variety of common evidence-based implementation science methodologies, the BHI team was able to rapidly implement an enduring set of telebehavioral health training series that may have helped many frontline mental health providers rapidly shift to providing telebehavioral health in response to the COVID-19 pandemic. By partnering with a variety of organizations, necessary supporting infrastructure and national access for these training series were achieved.

During of the initial onset of the pandemic, the BHI team had one goal: to deliver telebehavioral health trainings very quickly to support community-based frontline behavioral health providers with the tools and skills to be able to continue to provide services. In a very real way, both lives and livelihoods were at stake. Initial evaluation questions sought to identify further training and resource needs, which informed future training content. A confluence of factors—namely, the pandemic, the mandated telehealth training in Washington, and a relative vacuum of existing training specific to telebehavioral health—led to an unexpectedly high participation rate for the earlier training series. By utilizing the described implementation strategies and a series of PDSA cycles, the BHI team was able to adjust future trainings to meet the needs of this growing audience going forward.

As a result, the BHI team has been able to create progressive, relevant, practical, and enduring TeleBehavioral Health training series. These training series continue, and ongoing feedback continues to inform current and future TeleBehavioral Health training plans. We suggest those tasked with creating a training series of this magnitude consider using such a framework.

Using this approach and similar strategies, another project used a similar framework to implement a service-wide telebehavioral health curriculum to train a large mental health department (Felker et al., 2021). As the audience for this telebehavioral health training project was located at one facility, the training team was able to implement a more detailed and comprehensive training program that led to greater telebehavioral health skills and confidence in using telebehavioral health technologies to provide care. This curriculum went beyond the formal didactic stage reported here and added workplace learning and reflective practice. This training was completed just prior to the COVID-19 pandemic and helped the department rapidly transition to providing telebehavioral health care for their population.

Fortunately, advances are being made for training telebehavioral health in a variety of ways. Recommendations for developing telebehavioral health competencies include a longitudinal approach to training focusing on transition from training to practice, an evaluation of telebehavioral health skills/behaviors combined with evaluation of clinical care, and ongoing engagement with professional organizations and boards to move to a consensus (Hilty et al., 2018). Telesupervision is starting to be further evaluated. Initial findings suggest that compared to in-person supervision, there are no differences in satisfaction with the supervision process, working alliance, or perceived effectiveness, and that the same elements that create good supervision in-person are also important when providing telesupervision (Rousmaniere, 2014; Jordan & Shearer, 2019). Further suggestions for use of telesupervision include as follows: (1) establish a clear policy on use of telesupervision that adheres to the professional training organization, (2) screen trainees for their readiness to engage in supervision, and (3) assure that trainees have instruction on how to effectively use the chosen technology platform to include problem-solving skills (Jordan & Shearer, 2019).

## Resources

For further information about the BHI TeleBehavioral Health training series:

- Information about the TeleBH 101 and 201 webinar series can be found at https://bhinstitute.uw.edu/training-workforce-policy/training/past-presentations/.
- Information about the ongoing TeleBH 301 webinar series can be found at https://bhinstitute.uw.edu/training-workforce-policy/training/telebehavioral-health/.
- Information about the TeleBH 101 online series can be found at TeleBehavioral Health 101 Series (https://nrtrc.catalog.instructure.com/programs/telebehavioral-health-101-series) and for TeleBH 201 online at https://nrtrc.catalog.instructure.com/programs/telebehavioral-health-201.

For those interested in learning more about general telehealth training and education, additional resources include the National Consortium of Telehealth Resource Centers, including regional Telehealth Resource Centers and national Telehealth Resource Centers focused on technology and on policy (https://telehealthresourcecenter.org). The American Telemedicine Association (https://www.americantelemed.org) and various professional organizations including the American Psychiatric Association (https://www.psychiatry.org) and the American Psychology Association (https://www.apa.org) offer telehealth training materials and resources.

## Limitations

As this training was only designed as a broad-based lecture series, the study team was not able to assess how many providers and administrators changed their behaviors and started to implement telebehavioral health into their routine clinics, nor did this project assess confidence or competence developed because of these trainings. In addition, the online learning management software used has limitations, such as an inability to utilize pull down menus for registration responses, as well as a limitation on the number of questions, which impacted the ability to gather more information, such as location of services, and to collect the licensure, professional role, and special populations served data in a manner that is uniform and feasible for aggregation. Future evaluation and infrastructure plans will address these limitations. Lastly, over the course of the past 3 years, the expanding project team has been able to devote more time and resources to creating a more useful and informative website, to improved and more effective outreach and communications, and to more comprehensive data collection and analysis.

## Conclusion

In this commentary, the study team describes an enduring and broad-reaching telebehavioral health training program that is progressive and remained relevant to frontline mental health providers. This project was informed by common implementation science strategies that could serve as a model framework for other teams to use in creating training content to meet the needs of their trainees.

**Table 1:** Counts of unique learners and total participations for all training series, through June 2022

Series name	Dates	# of sessions	Unique learners	Total participations
Telehealth Provider Forum	Apr 2020–Jul 2021	18	665	1392
TeleBH 101 Webinar	Jan 2021–Feb 2021	6	2655	3825
TeleBH 201 Webinar	Oct 202–Sep 2021	12	517	786
TeleBH 301 Webinar	Jan 2022–Dec 2022 (ongoing)	6 of 12	664	1059
TeleBH 101 Online	Dec 2020–Jun 2022 (ongoing)	6	6796	12,079
TeleBH 201 Online	12-session series launched end of June 2022		N/A	N/A
Grand Total				19,141
